# Gender differences in clinical and psychosocial features in a large sample of Italian patients with schizophrenia

**DOI:** 10.1192/j.eurpsy.2022.2057

**Published:** 2022-09-01

**Authors:** P. Bucci, S. Galderisi, A. Rossi, P. Rocca, A. Bertolino, G.M. Giordano, A. Mucci, M. Maj

**Affiliations:** 1 University of Campania, Department Of Psychiatry, Naples, Italy; 2 University of L’Aquila, Department Of Biotechnological And Applied Clinicalsciences, Section Of Psychiatry, L’Aquila, Italy; 3 University of Turin, Department Of Neuroscience, Section Of Psychiatry, Turin, Italy; 4 University of Bari Aldo Moro, Basic Medical Science, Neuroscience And Sense Organs, Bari, Italy

**Keywords:** Gender differences, Recovery, schizophrénia, sex differences

## Abstract

**Introduction:**

An extensive literature regarding gender differences relevant to several aspects of schizophrenia is nowadays available. It includes some robust findings as well as some inconsistencies. The identification of gender differences and the understanding of their explanations may help to clarify the underlying etiopathogenetic mechanisms of specific aspects of the disorder.

**Objectives:**

The present study aimed at investigating gender differences on premorbid, clinical, cognitive and outcome indices, as well as their impact on recovery, in a large sample of patients with schizophrenia recruited within the multicenter study of the Italian Network for Research on Psychoses.

**Methods:**

State-of-the-art instruments were used to assess the investigated domains. Group comparisons between male and female patients were performed on all considered indices. The associations of premorbid, clinical and cognitive indices with recovery in the two patient groups were investigated by means of multiple regressions.

**Results:**

Males with respect to females had a worse premorbid adjustment – limited to the academic dimension – an earlier age of onset, a higher frequency of history of substance and alcohol abuse, more severe negative symptoms (both avolition and expressive deficit), positive symptoms and impairment of social cognition. No gender difference was observed in neurocognition nor in the rates of recovery.

**Conclusions:**

Although males showed some disadvantages in the clinical picture, this was not translated into a worse outcome. This finding may be related to the complex interplay of several factors acting as predictors or mediators of outcome.

**Disclosure:**

No significant relationships.

